# Activation of iNKT Cells Prevents Salmonella-Enterocolitis and Salmonella-Induced Reactive Arthritis by Downregulating IL-17-Producing γδT Cells

**DOI:** 10.3389/fcimb.2017.00398

**Published:** 2017-09-08

**Authors:** Mariángeles Noto Llana, Sebastián H. Sarnacki, Andrea L. Morales, María del R. Aya Castañeda, Mónica N. Giacomodonato, Guillermo Blanco, María C. Cerquetti

**Affiliations:** ^1^Departamento de Microbiología, Parasitología e Inmunología, Facultad de Medicina, Universidad de Buenos Aires Buenos Aires, Argentina; ^2^Instituto de Investigaciones en Microbiología y Parasitología Médica, CONICET, Universidad de Buenos Aires Buenos Aires, Argentina; ^3^Departamento de Inmunología, Facultad de Farmacia y Bioquímica, Universidad de Buenos Aires Buenos Aires, Argentina; ^4^Instituto de Estudios de la Inmunidad Humoral, CONICET, Universidad de Buenos Aires Buenos Aires, Argentina

**Keywords:** *Salmonella enterica*, enterocolitis, γδT cells, IL-17, mice, joint inflammation

## Abstract

Reactive arthritis (ReA) is an inflammatory condition of the joints that arises following an infection. *Salmonella* enterocolitis is one of the most common infections leading to ReA. Although the pathogenesis remains unclear, it is known that IL-17 plays a pivotal role in the development of ReA. IL-17-producers cells are mainly Th17, iNKT, and γδT lymphocytes. It is known that iNKT cells regulate the development of Th17 lineage. Whether iNKT cells also regulate γδT lymphocytes differentiation is unknown. We found that iNKT cells play a protective role in ReA. BALB/c Jα18^−/−^ mice suffered a severe *Salmonella* enterocolitis, a 3.5-fold increase in IL-17 expression and aggravated inflammation of the synovial membrane. On the other hand, activation of iNKT cells with α-GalCer abrogated IL-17 response to *Salmonella* enterocolitis and prevented intestinal and joint tissue damage. Moreover, the anti-inflammatory effect of α-GalCer was related to a drop in the proportion of IL-17-producing γδT lymphocytes (IL17-γδTcells) rather than to a decrease in Th17 cells. In summary, we here show that iNKT cells play a protective role against *Salmonella*-enterocolitis and *Salmonella*-induced ReA by downregulating IL17-γδTcells.

## Introduction

After infections of the gastrointestinal system a spondyloarthropathy which includes joint inflammation can arise. This alteration is known as Reactive arthritis (ReA) (Townes, 2010). ReA is predominant in poor countries with high proportions of gastrointestinal infections; 30% of the cases are associated to *Salmonella*- induced enterocolitis (Sinha et al., 2003; World Health Organization, Food Safety and Foodborne Illness, 2007). Earlier we have described an animal model for *Salmonella* enterocolitis in which soon after onset of infection, mice develop joint lesions (Noto Llana et al., 2009). During *Salmonella* enterocolitis, the increase in IL-17 expression is closely associated with both intestinal and joint damage (Noto Llana et al., 2012). Moreover, when IL-17 expression is downregulated the intestinal and joint tissue injury is prevented (Noto Llana et al., 2013). Although the pathogenesis of ReA remains unclear, IL-17 generated in intestine and mesenteric lymph nodes plays a central role in the initial stages of this process (Noto Llana et al., 2012).

IL-17-producers cells are mainly Th17, iNKT, and γδT lymphocytes. NKT cells are a subset of T lymphocytes that express NK cell markers. Although not very numerous, the distinctive properties of iNKT cells make them important in modulating a variety of other cell types including myeloid cells, NK cells, and cells of the adaptive immune system (Van Kaer et al., 2013). In mice, the majority of NKT cells express an invariant T cell receptor (TCR) encoded by Vα14Jα18 gene segment. The receptor recognizes glycolipid antigens presented by CD1d, a non-classical antigen-presenting molecule. Stimulation of TCR induces iNKT cells to rapidly secrete large amounts of IL-17 (Van Kaer, 2005). It has been shown that the absence of iNKT cells leads to an altered gut microbiota, with increased number of inflammatory bacterial species; interesting there is also evidence that intestinal microbiota can control iNKT cell function upon activation during gut inflammation (Selvanantham et al., 2016). The role of iNKT cells in *Salmonella*- induced intestinal inflammation is still to be elucidated.

*Salmonella* infection induces activation of adaptive and T cells, after which IL-17 expression appears (Godinez et al., 2009). Moreover, through *Salmonella* infection, developed Th17 cells perform a significant role in host intestine defense (McGeachy and McSorley, 2012). In turn, iNKT cells have been indicated to limit the development of Th17 lineage and to provide a natural barrier against Th17 responses (Mars et al., 2009). Whether Th17 represent a significant source of IL-17 during *Salmonella* intestinal infection remains unknown.

Based on their properties, γδ lymphocytes are considered as specific T cells that regulate immune response. Signals from the environment are detected and sensed by γδT cells; after that they are able to initiate immune-surveillance at the site. They produce proinflammatory cytokines, such as IL-17, and activate adaptive immune cells (Patil et al., 2015).

In this work, we found that the downregulation of IL-17-producing γδT cells (IL17-γδTcells) by iNKT cells prevents *Salmonella*-enterocolitis and ReA. Understanding the mechanisms that regulate γδ T cell functions would be useful for developing treatment and prevention strategies for numerous diseases, including ReA.

## Materials and methods

### Mice

BALB/c Jα18^−/−^ mice were kindly provided by Dr. Masaru Taniguchi (Riken, Yokohama, Japan), wild type (WT) BALB/c mice were obtained from our vivarium; animals maintained in a germ-free atmosphere and in accordance with the guidelines of the NIH (Guide for the Care and Use of Laboratory Animals: 8th edition 2011. p. 120–124). Animals were provided with food and water *ad libitum*. All procedures were approved by the Internal Committee for the Care and Use of Laboratory Animal (CICUAL) from the School of Medicine, University of Buenos Aires (Res. CD 2950/2013).

### Bacterial strain and growth condition

*Salmonella enterica* serovar Enteritidis #5694 was used to infect mice. Bacteria were cultured in trypticase soy broth at 37°C, 200 cycles per minute, pelleted by centrifugation and suspended to the appropriate density in saline. The number of bacteria was determined by plating appropriate dilutions on trypticase soy agar plates.

### *Salmonella* infection and generation of enterocolitis (EC)

Twenty-four hours before infection mice were given 20 mg of streptomycin intragastrically, For intragastric inoculation, 0.2 ml of the bacterial suspension (3–4 × 10^3^ CFU) was introduced into the stomach with a 21 G blunt needle on a 1.0 ml plastic syringe.

### Bacterial colonization

Mice were sacrificed at different time-points, spleens were removed aseptically, and homogenized in saline. Samples were diluted appropriately in saline and plated on *Salmonella*-*Shigella* (SS) agar.

### Reagents and monoclonal antibodies

For activation of iNKT cells, 2 h before infection mice received 4 μg of α-*galactosylceramide* (α-GalCer; Pharmaceutical Research Laboratories, Japan) intraperitoneally (i.p.). α-GalCer solution (0.2 mg/ml) was prepared in 0.5% polisorbate and conserved at −20°C (Bharhani et al., 2009).

Monoclonal anti-CD1d and the isotype-matched control Ig were obtained from BD Biolegend (clone 1B1). For blocking experiments, mice were injected i.p. with 100/100 μg of mAb 15 min before bacterial challenge (Jones et al., 2009). For depletion of γδT cells we used functional-grade purified anti-mouse TCRγδ mAb (UC7-13D5) obtained from eBioscience. Mice were injected i.p. with 200 μg of anti-mouse TCRγδ 3 days before *Salmonella* infection (Li et al., 2012).

### Pathology

Intestinal samples (ileum) were fixed in formalin and processed by standard procedures for paraffin embedding (Noto Llana et al., 2012). Knee joints were dissected, fixed in formalin for 2 days, decalcified in EDTA for 30–40 days, and then embedded in paraffin. Standard sections of 5 μm were prepared and stained with haematoxylin–eosin (HE). To quantify the severity of the pathologic changes, we used a scoring system devised by Noto Llana et al. (2009).

### RNA purification and RT-qPCR

Total RNA was extracted and cDNA was synthesized as described previously (Noto Llana et al., 2013). The primers used to determine the expression of IL-17, VαJα, and IFN-γ were described previously (Kim et al., 2005; Godinez et al., 2009; Nur et al., 2013) and are shown in Supplementary Table 1. qPCR was performed using SyBr Green PCR kit (Applied Biosystems Inc., Foster City, CA) in an Applied Biosystems 7500 sequence detector. Relative expression levels were normalized to 18S.

### Flow cytometry

Single-cell suspensions of lymph nodes were prepared by mashing the organs through a stainless steel mesh. Single cell suspensions were washed and suspended in PBS. One hundred thousand cells were stained with Biolegend antibodies. For extracellular stain anti-CD4 (Alexa Fluor® 488), anti-CD3 (PE/Cy7), and anti-TCR γδ (FITC) were used in 100 μl of PBS for 30 min at 4°C. For intracellular staining, cells were incubated in RPMI with gentamicin 40 mg/ml and 10% FBS, stimulated with 25 ng/ml PMA, 500 ng/ml ionomycin, and 10 μg/ml BFA (BD Golgi Plug) for 24 h at 37°C in 5% CO_2_. Cells were fixed in 4% paraformaldehyde and permeabilized with Perm Wash (BD Biosciences). Finally, cells were stained using anti-IL-17 (PE) and anti-IFNγ (APC) in 100 μl of PBS for 30 min at 4°C, and washed with PBS. Samples were analyzed using a PASIII flow cytometer and data acquired were processed using WinMDI software.

### Statistical analysis

Unless otherwise stated, all results are the average ± *SD* from at least three separate experiments. *P*-values were determined using Mann Whitney test for non-parametric values. *p* < 0.05 was considered to be statistically significant.

## Results

### iNKT KO mice suffer severe *Salmonella* enterocolitis and joint inflammation

*Salmonella* enterocolitis initiates early joint inflammation. We have previously demonstrated that IL-17 plays a crucial role in this phenomenon (Noto Llana et al., 2012). To examine the involvement of iNKT cells in this event, *Salmonella* enterocolitis (EC) was induced in Jα18^−/−^ mice (KO EC group) as well as in WT mice (EC group). Five days after infection intestinal and knee samples were studied.

**Figure 1:**
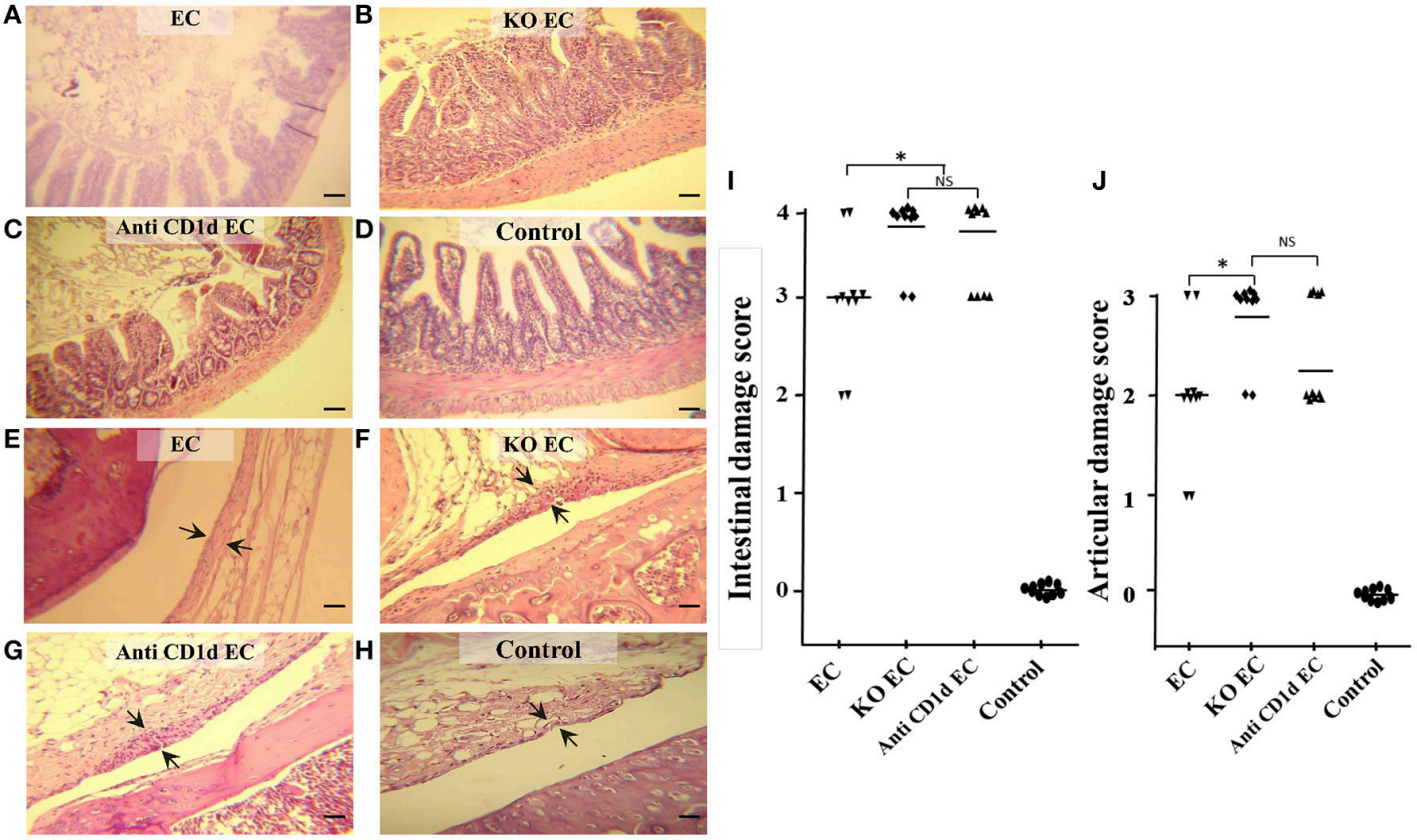
Lack of iNKT aggravates *Salmonella* enterocolitis and *Salmonella*-induced ReA. Wild type and Jα18^−/−^ KO BALB/c mice pretreated with 20 mg of streptomycin received 3–4 × 10^3^ CFU of *Salmonella enterica* intragastrically. Samples were taken 5 days after bacterial inoculation. Representative HE sections of intestine **(A–D)** and knee joint **(E–H)**. Uninfected wild type mice, *Control group* **(D,H)**; infected wild type mice, *EC group*, **(A,E)**; infected KO mice, *KO EC group* **(B,F)**; infected wild type mice treated with anti-CD1d mAb, *anti-CD1d EC group* **(C,G)**. Scale bar 100 m. Arrows indicate synovial membrane. Differences in intestinal damage scores **(I)**. Differences in joint damage scores **(J)**. ^*^Significant differences (*p* < 0.05). NS, no significant differences. Data were collected from three independent experiments. No differences in histology scores were found between uninfected KO mice or uninfected WT mice treated with anti-CD1d and Control mice.

KO EC animals developed a more severe intestinal inflammation compared with WT infected mice (EC). KO EC animals showed loss of mucosal integrity, intestinal epithelium diminished in height and absorptive villi thickened and fused together (Figure 1B). Diffuse mild enteritis was present in WT animals (EC group); absorptive villi were thickened and fused together but epithelial barrier was conserved (Figure 1A). In consonance, intestinal histological score in KO mice was significantly higher (*p* < 0.05) when compared with WT mice (KO EC vs. EC; Figure 1I).

**Figure 2:**
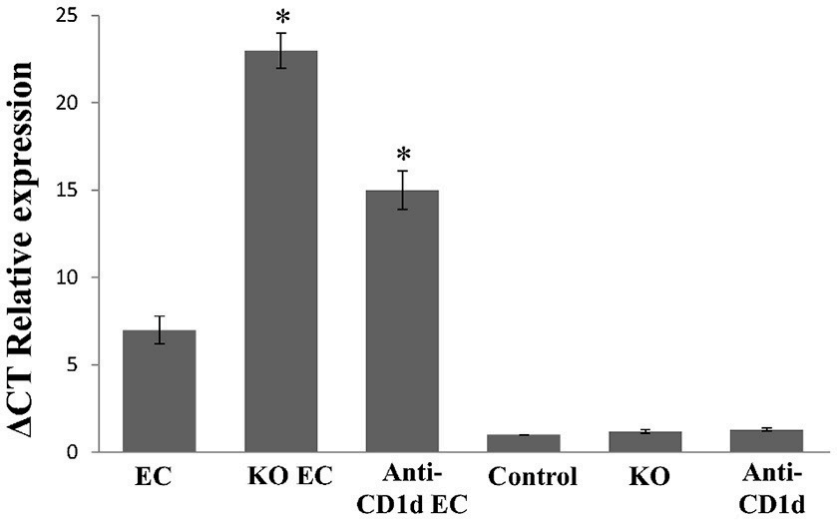
Augmented IL-17 expression in mesenteric lymph nodes after *Salmonella* enterocolitis onset. Wild type and Jα18^−/−^ KO BALB/c mice pretreated with 20 mg of streptomycin received 3–4 × 10^3^ CFU of *Salmonella enterica* intragastrically. Five days after infection cytokine expression was analyzed by qPCR. Infected wild type mice (*EC group*); infected KO mice (*KO EC group*); infected wild type mice treated with anti-CD1d mAb (*anti-CD1d EC group*); uninfected wild type mice *(Control group)*; uninfected KO mice *(KO group)*; uninfected wild type animals treated with anti-CD1d mAb *(anti-CD1d group)*. Expression levels for mRNA were determined and are presented in relation to 18S rRNA. ^*^Significant differences between *KO EC* or *anti-CD1d EC* mice vs. *EC group* (*p* < 0.01). Results are expressed as mean ± *SD* (*n* = 4). Representative data from three independent experiments.

IL-17 is among the most prominent inflammatory cytokines secreted soon after Salmonella infection (Godinez et al., 2009), and it is involved in the initiation of joint inflammation (Noto Llana et al., 2012, 2013). Therefore, we analyzed IL-17 expression in mesenteric lymph nodes (MLN) of animals lacking iNKT cells infected with Salmonella. We found that, in the absence of iNKT, IL-17 expression increases. As shown in Figure 2, a 3.5-fold increase was detected in KO mice 5 days after the onset of enterocolitis (KO EC vs. EC; *p* < 0.01). Concomitantly, KO EC group also showed moderate to severe hyperplasia of synovial membrane, consistent with a score of 3 [Figure 1F (arrows) and Figure 1J]. WT animals receiving *Salmonella* (EC group) presented mild hyperplasia of the synovial membrane [Figure 1E (arrows) and Figure 1J, score 1–2].

Participation of iNKT cells during *Salmonella* enterocolitis was also analyzed using anti-CD1d antibodies in mice suffering enterocolitis (anti-Cd1d EC group). Intestinal and synovial histology as well as MLN IL-17 expression in these animals were similar to that observed in infected animals lacking iNKT (anti CD1d EC vs. KO EC; Figures 1, 2). No differences in histology scores or IL-17 expression were found between infected mice treated with Ig isotype control and EC group (not shown). Altogether, these findings suggest that iNKT cells play an active role in protecting the host from *Salmonella* enterocolitis by decreasing IL-17 expression.

### Th17 cells are not the main source of IL-17 generated during *Salmonella* enterocolitis

**Figure 3:**
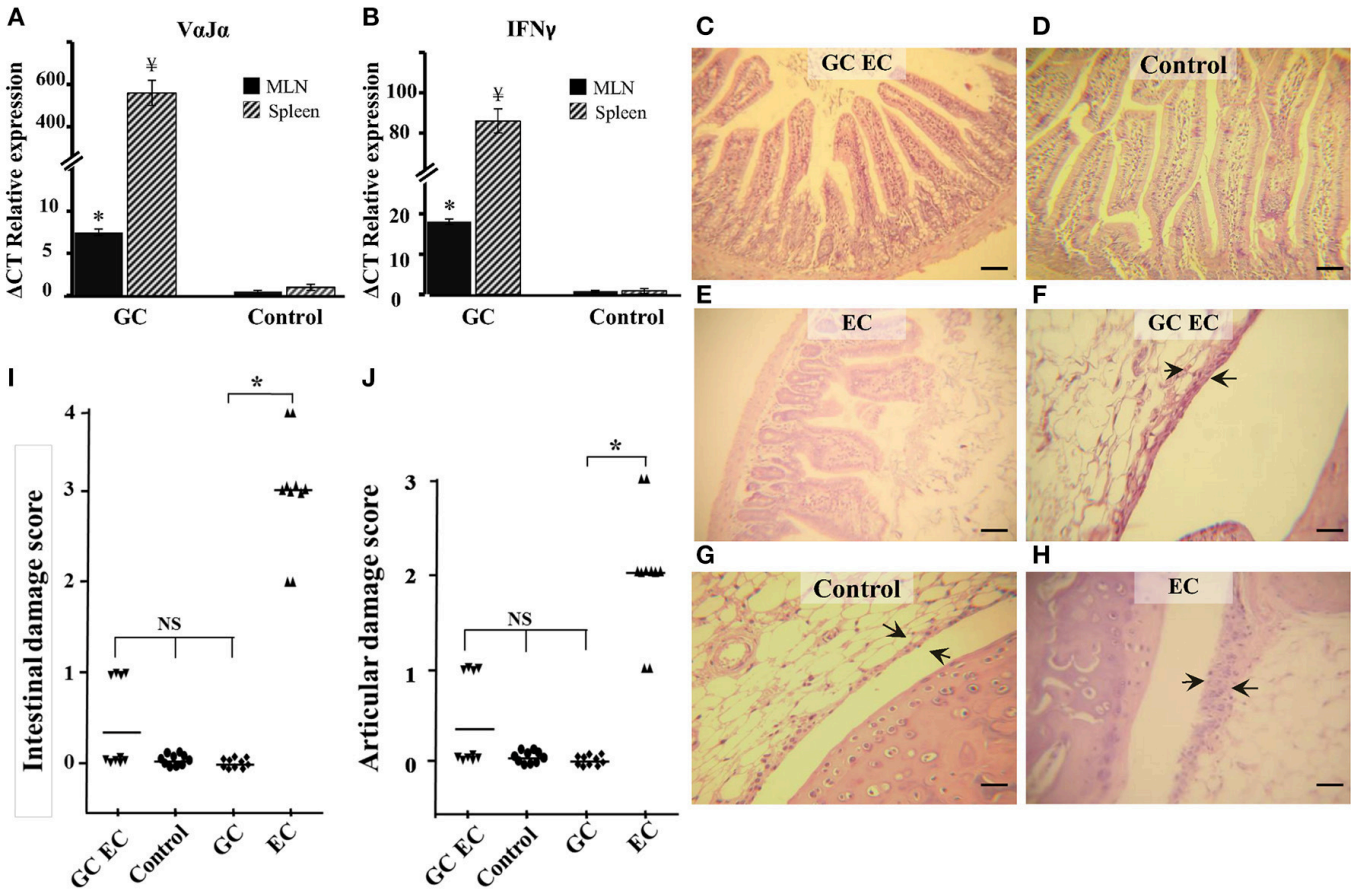
α-GalCer treatment prevents intestinal and joint inflammation induced by *Salmonella* enterocolitis. Wild type BALB/c mice pretreated with 20 mg of streptomycin received 3–4 × 10^3^ CFU of *Salmonella*. Samples were taken 5 days post-infection. VαJα **(A)** and IFN-γ **(B)** expression in mesenteric lymph nodes (MLN; black solid) and spleen (striped) analyzed by qPCR. ^*^ ¥Significant differences between α-GalCer-treated mice (*GC group*) and untreated mice (*Control group*); *p* < 0.01. Results are expressed as mean ± *SD* (*n* = 4). Representative data from three independent experiments. Representative HE sections of intestine **(C–E)** and knee joint **(F–H)**. Uninfected mice, *Control group* **(D,G)**; infected mice treated with α-GalCer, *GC EC group* **(C,F)**; infected mice, *EC group* **(E,H)**. Arrows indicate synovial membrane. Scale bar 100 um. Differences in intestinal damage scores **(I)**. Differences in joint damage scores **(J)**. NS, no significant differences. Data were collected from three independent experiments. ^*^Significant differences (*p* < 0.05).

**Figure 4:**
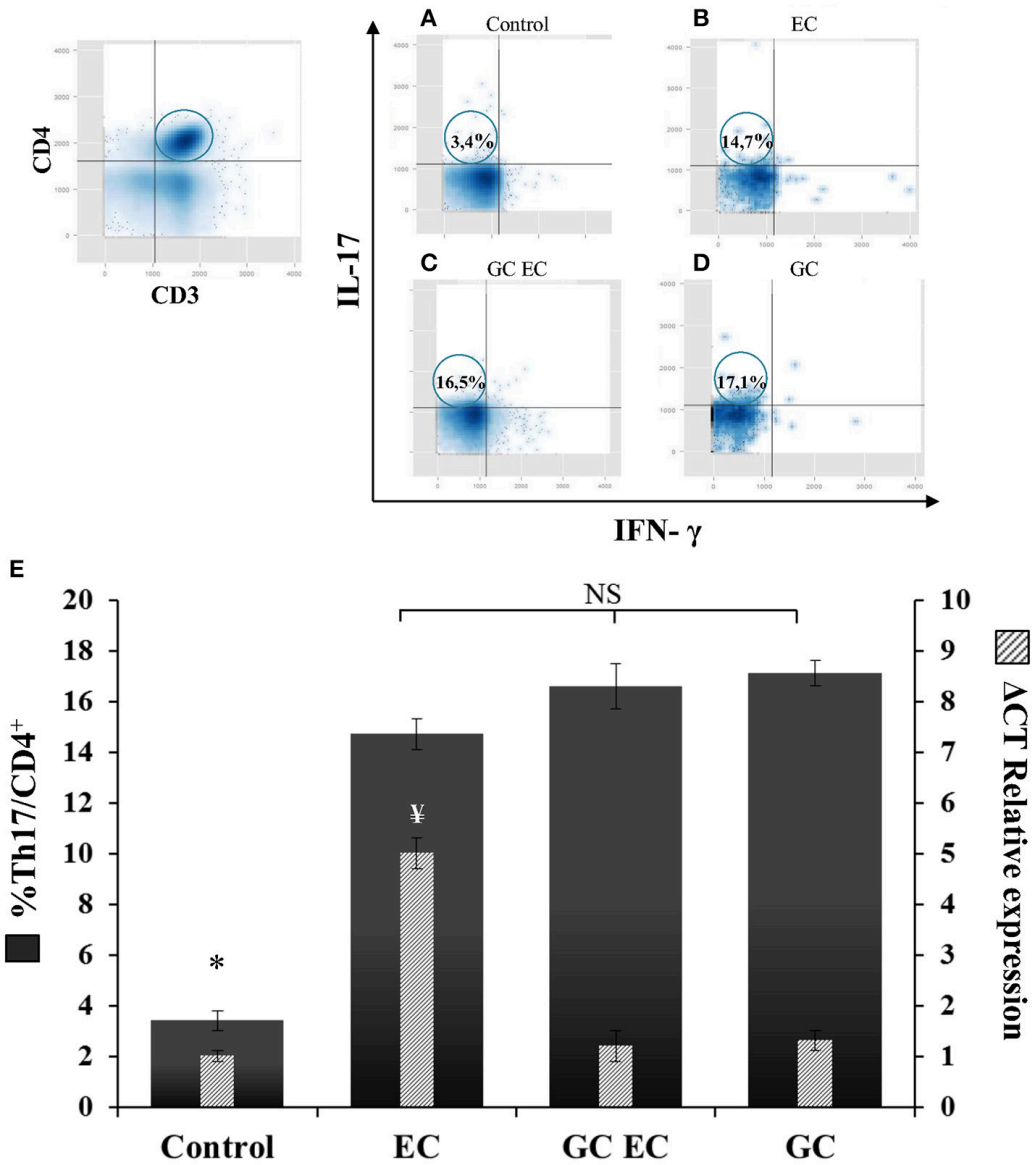
α-GalCer treatment increases the percent of Th17 cells and reduces IL-17 production in mesenteric lymph nodes. Wild type BALB/c mice pretreated with 20 mg of streptomycin received 3–4 × 10^3^ CFU of *Salmonella*. Samples were taken 5 days post-infection. Flow cytometry. The number of Th17 cells was identified by CD3^+^CD4^+^ double positive cells and gated by intracellular staining of IFN-γ^−^ IL-17^+^ (*left panel*). Uninfected mice, *Control group* **(A)**; infected mice, *EC group* **(B)**; infected mice treated with α-GalCer, *GC EC group* **(C)**; uninfected mice treated with α-GalCer, *GC group* **(D)**. Percentage of Th17 cells (solid bars) and IL-17 expression in mesenteric lymph nodes (stripped bars) **(E)**. Expression was determined by qPCR, levels for mRNA were determined and are presented in relation to 18S rRNA. Results are expressed as mean ± SD (*n* = 4). ^*^Significant differences in the percentage of Th17 cells with respect to the other three groups (*p* < 0.05); NS, no significant differences. ^¥^Significant differences in IL-17 expression with respect to the other three groups (*p* < 0.05). Representative data from three independent experiments.

We next investigated the influence of iNKT cells stimulation on the course of *Salmonella* infection. Mice were injected with α-GalCer in order to activate iNKT cells prior to *Salmonella* uptake. α-GalCer treatment (GC group) resulted in a significant increase of VαJα and IFN-γ (Figures 3A,B). IFN-γ was determined as its expression is indicative of iNKT cell activation. We found that α-GalCer treatment attenuates the inflammatory host response to the pathogen. Studies performed 5 days after enterocolitis onset showed no histological differences between the intestines from infected animals treated with α-GalCer (GC EC group) and uninfected controls (Figures 3C,D,E,I). At the same time point, we analyzed bacterial colonization of spleen and survival in mice receiving *Salmonella*. Our results indicated that there were no significant differences in splenic bacterial colonization and mice survival between animals with enterocolitis (EC) and those who received GC before infection (GC EC; data not shown). Next, we analyzed whether the beneficial effect of iNKT activation was related to a down regulation of IL-17 production. Therefore, IL-17 expression and Th17 cell population were tested in MLN of infected mice treated with α-GalCer. CD3^+^CD4^+^ cells were gated, then intracellular staining for IL-17^+^ and IFN-γ^−^ was considered to be Th17 cells (Figures 4A–D). During *Salmonella* enterocolitis (EC group) IL-17 expression increased five times compared with uninfected animals, with a concomitant 11% increase in Th17 cell population (Figure 4E). Interestingly, α-GalCer treatment reduced the expression of IL-17 in infected mice (GC EC group) down to control levels without reducing the number of Th17 cells (Figure 4E). In uninfected animals α-GalCer treatment induced an increase in Th17 cells without a significant increment in IL-17 expression (GC group, Figure 4E). Altogether, these results indicate that the beneficial effect of α-GalCer treatment on *Salmonella* enterocolitis could be related to a decrease in the expression of IL-17 in cells other than Th17 or iNKT, such as γδT cells.

### Activation of iNKT cells downregulates IL17-γδTcells

**Figure 5:**
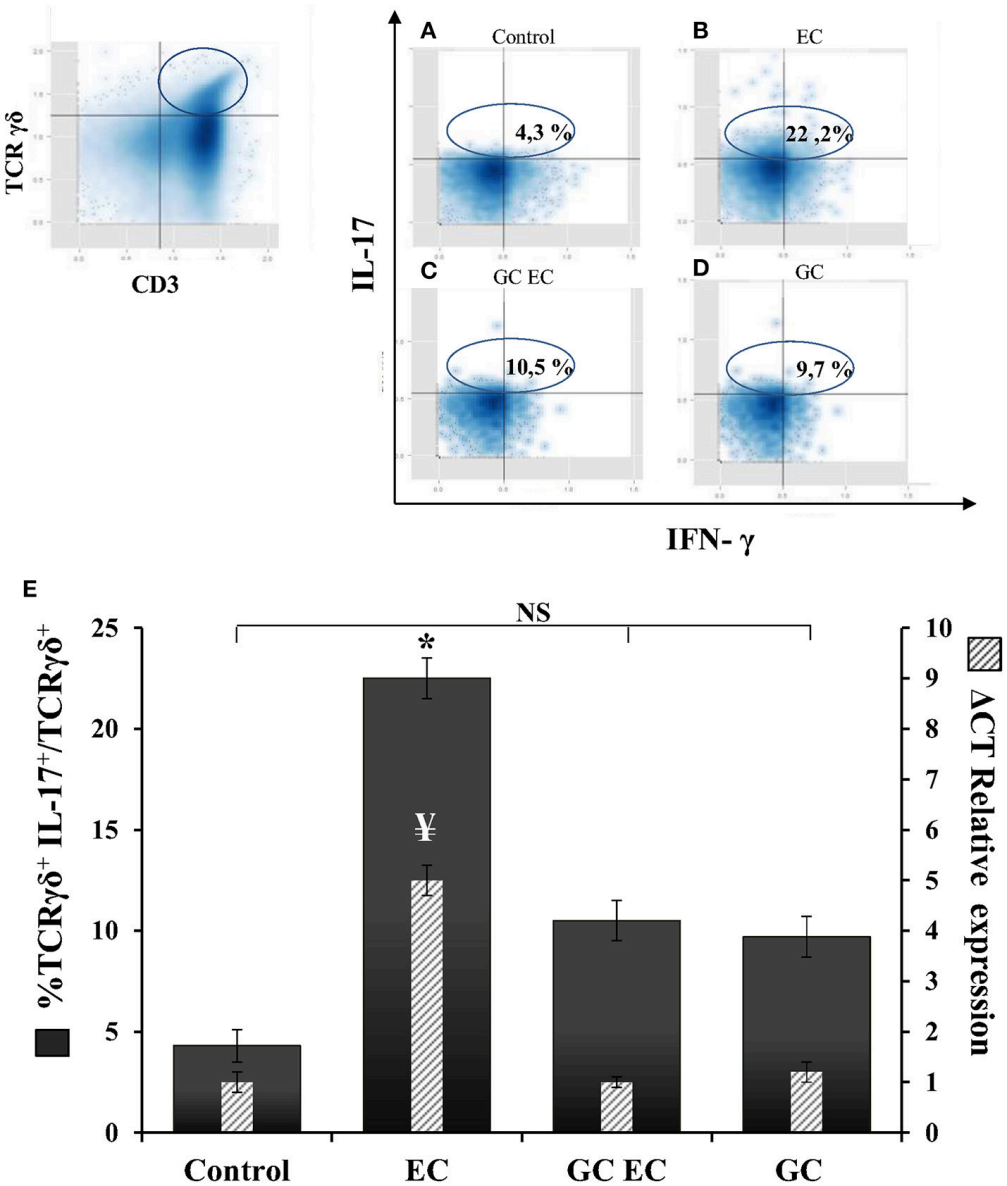
α-GalCer treatment decreases the percent of TCRγδ^+^IL-17^+^ cells and reduces IL-17 production in mesenteric lymph nodes. Wild type BALB/c mice pretreated with 20 mg of streptomycin received 3–4 × 10^3^ CFU of *Salmonella*. Samples were taken at day 1 post-infection. Flow cytometry. The number of TCRγδ^+^IL-17^+^ cells was identified by CD3^+^TCRγδ^+^ double positive cells and gated by intracellular staining of IL-17^+^ (*left panel*). Uninfected mice, *Control group* **(A)**; infected mice, *EC group* **(B)**; infected mice treated with α-GalCer, *GC EC group* **(C)**; uninfected mice treated with α-GalCer, *GC group* **(D)**. Percentage of TCRγδ^+^IL-17^+^ cells (solid bars) and IL-17 expression in mesenteric lymph nodes (stripped bars) **(E)**. Expression was determined by qPCR, levels for mRNA were determined and are presented in relation to 18S rRNA. Results are expressed as mean ± *SD* (*n* = 4). ^*^Significant differences in the percentage of TCRγδ^+^IL-17^+^ cells with respect to the other three groups (*p* < 0.05); NS, no significant differences. ^¥^Significant differences in IL-17 expression with respect to the other three groups (*p* < 0.05). Representative data from three independent experiments.

γδT cells contribute to the IL-17 production during murine salmonellosis (Godinez et al., 2009) hence, we next analyzed whether the protective effect of α-GalCer involves down regulation of these cells. Flow cytometry analysis of γδT cells and IL-17 expression were assessed in MLN 1 day after *Salmonella* infection in mice with and without α-GalCer treatment. CD3^+^ TCRγδ^+^ IL-17^+^ cells were considered as IL17-γδTcells (Figures 5A–D). In infected animals without α-GalCer treatment (EC group) 22.2% of TCRγδ^+^ cells are IL-17^+^ producers whereas in uninfected mice (Control group) these cells represent only 4.3% (Figure 5A). α-GalCer treatment in infected mice (GC EC group) significantly reduced the percentage of IL17-γδTcells to 10.5% (Figure 5C). Concomitantly in this group the expression of IL-17 in MLN was significantly diminished compared to the enterocolitis group (GC EC vs. EC, Figure 5E). Therefore, the beneficial effect of α-GalCer treatment on *Salmonella*-induced intestinal inflammation could be attributed to a reduction in the percentage of IL17-γδTcells. In addition, we found that activation of iNKT prevented *Salmonella*- induced joint inflammation (Figures 3F,G,H,J).

**Figure 6:**
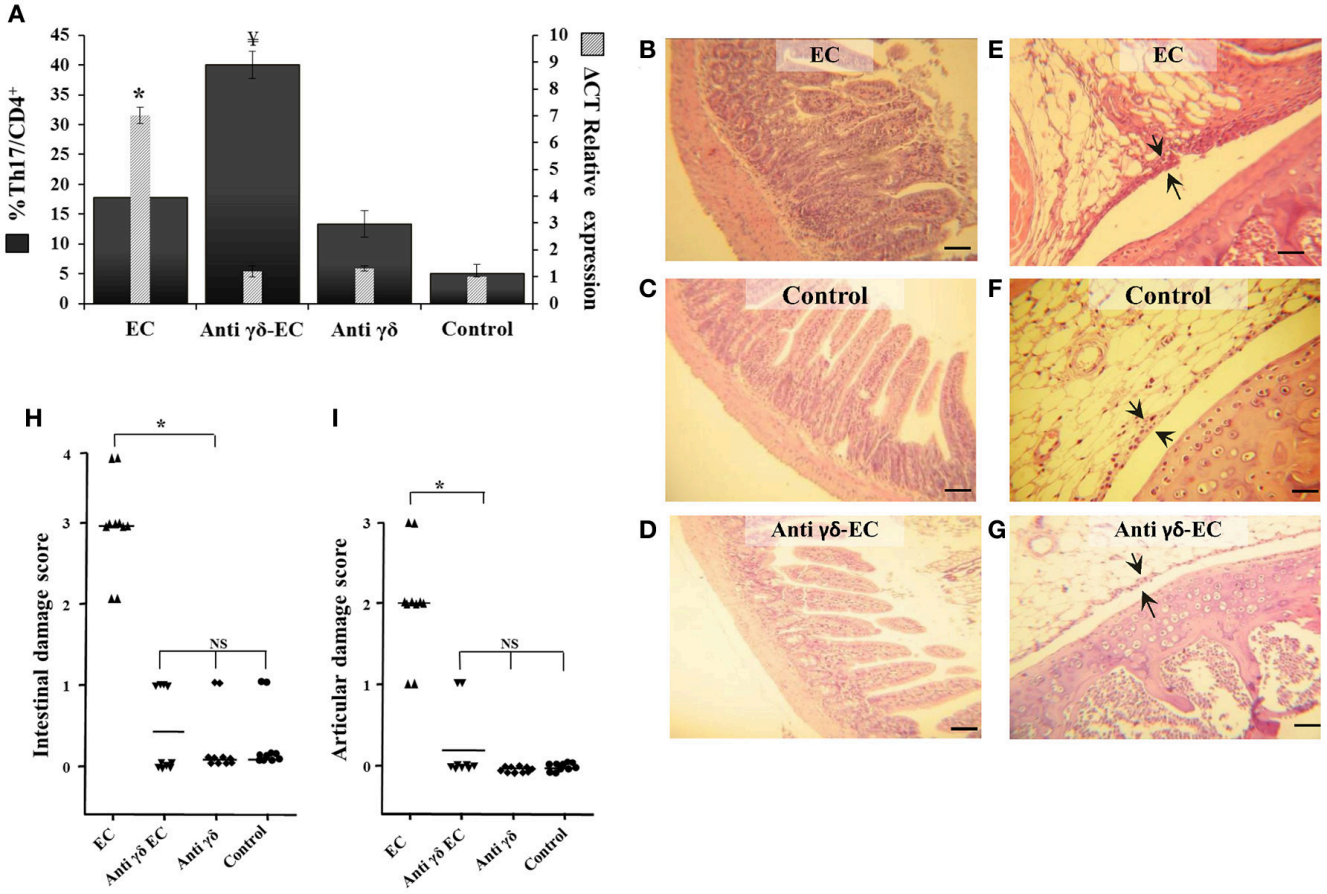
Depletion of TCRγδ cells reduces IL-17 expression, increases Th17^+^ total cell number and prevents intestinal and joint inflammation induced by *Salmonella* enterocolitis. Wild type BALB/c mice pretreated with 20 mg of streptomycin received 3−4 × 10^3^ CFU of *Salmonella*. Samples were taken 5 days post-infection. Flow cytometry. The number of Th17 cells was identified by CD3^+^CD4^+^ double positive cells and gated by intracellular staining of IFN-γ^−^ IL-17^+^. Percentage of Th17 cells (solid bars) and IL-17 expression in mesenteric lymph nodes (stripped bars) **(A)**. Interleukin expression was determined by qPCR, levels for mRNA were analyzed by qPCR in mesenteric lymph nodes and are presented in relation to 18S rRNA. *EC group*: mice suffering from enterocolitis, *Anti*-γδ *EC*: *Salmonella* infected mice treated with anti-TCRγδ mAb. *Anti*-γδ *group*: uninfected animals treated with anti-TCRγδ mAb. *Control group*: uninfected untreated mice. ^¥^Significant differences in the percentage of Th17 cells with respect to the other three groups (*p* < 0.05). ^*^Significant differences in IL-17 expression with respect to the other three groups (*p* < 0.01); NS, no significant differences. Results are expressed as mean ± *SD* (*n* = 4). Representative data from 3 independent experiments. Representative HE sections of intestine **(B–D)** and knee joint **(E–G)**. *EC group* **(B,E)**. *Anti*-γδ *EC group* **(D,G)**; *Control group* **(C,F)**. Arrows indicate synovial membrane. Scale bar 100 m.

Additional experiments were performed using specific mAb anti TCRγδ. Three days after treatment the number of TCRγδ cells was reduced in about 55% (data not shown). As shown in Figure 6, blockage of TCRγδ cells significantly reduced the expression of IL-17 in the MLN of *Salmonella* infected animals (anti-γδ EC group, Figure 6A). Depletion of TCRγδ cells also ameliorated intestinal and joint inflammation induced by enterocolitis (Figures 6D–G); in both cases, histological scores dropped to 0 resembling those of uninfected mice (Figures 6H,I). No histological changes were observed in uninfected mice treated with anti-TCRγδ mAb (data not shown). These results suggest that γδT cells play an active role in *Salmonella*-induced intestinal inflammation and ReA sequelae.

Interestingly, we have found that depletion of TCRγδ cells results in a 2-fold increase of Th17 cells in mice suffering enterocolitis compared to infected animals without TCRγδ cell depletion (Figure 6A). Moreover, depletion treatment induces an augment in Th17 cells in animals without bacterial infection compared to control mice (17 vs. 5%). Further investigation is needed to better understand the role of TCRγδ cells in regulating Th17 cells.

## Discussion

Upon *Salmonella* infection, IL-17 can be primarily produced by Th17, γδ, and NKT cells existent in the intestinal tissue (Schulz et al., 2008; Godinez et al., 2009). These cells have been also suggested as the connection between both gut and joint inflammation during arthritis, as they are involved in IL-17 expression (Al-Mossawi et al., 2013). iNKT cells can exhibit pro-inflammatory and anti-inflammatory characteristics; depending on how they are activated, and the signals present in the resident environment (O'Keeffe et al., 2015). Therefore, iNKT cells can have either protective or harmful roles in many pathological states. In fact, in mice, iNKT cells are known to promote a diversity of autoimmune arthritis (Chiba et al., 2005).

Here, we used the murine model for enterocolitis and ReA to determine the role of iNKT in the intestinal and joint inflammatory response to *Salmonella*. KO mice and anti-CD1d-treated animals presented a more severe intestinal and joint damage suggesting that iNKT cells play a protective role. Experiments involving specific activation of iNKT cells support this hypothesis since *Salmonella*-infected mice receiving α-GalCer treatment showed no sign of gastrointestinal or joint disease. Using an animal model of intra-articular infection, Bharhani et al. showed that the activation of iNKT cells confers protection against *Chlamydia trachomatis*-induced arthritis (Bharhani et al., 2009). Authors showed that α-GalCer treatment protects the host in the early phase of the arthritis and also ameliorates the established disease; the mechanism of protection induced by iNKT cells was not determined.

We show that the lack of iNKT cells during *Salmonella* enterocolitis triggers an increase of IL-17 expression and concomitantly induces a severe damage in intestinal and synovial tissues. In contrast, activation of iNKT cells protects the host from gut and joint inflammation and averted the early IL-17 response to *Salmonella* infection in MLN.

Soon after *Salmonella* infection, activation of adaptive and T cells occurs, which induces IL-17 expression (Godinez et al., 2009). In the intestine response to this pathogen, Th17 cells are key players defending from infection (McGeachy and McSorley, 2012). On the other hand, iNKT cells have been indicated to limit the development of Th17 lineage and to provide a natural barrier against Th17 responses (Mars et al., 2009). Within this context we speculated that the protective effect of iNKT activation on *Salmonella*-induced inflammation could be attributed to a down regulation of Th17 cells and to the concomitant reduction in IL-17 expression. Our results showed that iNKT activation reduces IL-17 expression in MLN of infected animals; notwithstanding, Th17 cells were not down regulated. Moreover, the percentage of Th17 cells after α-GalCer treatment was significantly increased in MLN.

We focused then on γδT cells as they contribute to the IL-17 produced early after *Salmonella* infection (Godinez et al., 2009). γδT cells constitute a cell population that is related to innate and adaptive immunity and is responsible for producing cytokines after injury. Secreted cytokines define their properties. The principal cytokine produced by γδT cells in humans is IFNγ, with an anti-viral, anti-bacterial, and anti-tumor immunity role (Patil et al., 2015). However, they can also be skewed toward IL-17, IL-4, or TGF-β producing phenotype (Caccamo et al., 2013). Numerous researches in recent years have described the role of Tγδ17 cells in bacterial infection, inflammatory disease, and cancer (Peng et al., 2008; Wakita et al., 2010; Cai et al., 2011). In this work we found that as early as 24 h post-infection *Salmonella* induces an increase in the expression of IL-17 concomitant with an augmentation of Tγδ17 in MLN. In line with these results, it has been reported that Tγδ17 are the primary source of IL-17 in early disease condition and play a significant role in regulating inflammation (Patil et al., 2015). Moreover, as shown in collagen-induced arthritis in mice, the Tγδ17 cell population expands related to severity of the pathology (Ito et al., 2009). α-GalCer treatment prior to the onset of *Salmonella* enterocolitis resulted in a significant reduction of both IL-17 expression and Tγδ17 cells and prevented synovitis. In agreement, it has been reported that activation of iNKT cells, using a synthetic analog of α-GalCer, suppresses murine collagen-induced arthritis (Yoshiga et al., 2011). In this model, the suppressive effect was dependent on IFN-γ induced by NKT cells. In our work, despite the drop of Tγδ17 cells percentage observed in α-GalCer treatment in animals with enterocolitis, mice receiving α-GalCer treatment alone showed an increase in Tγδ17 cells percentage comparing to control untreated mice. This effect could be related to the fact that γδ T cells recognize lipids presented by non-classical MHC molecules, such as molecules from the CD1 family and can also be activated during the early stages of bacterial infection. Both phenomena are also observed in MAIT cells (mucosal associated invariant T cells) which together with Tγδ17 and iNKT cells are the major populations within the innate T cell group (Gao and Williams, 2015).

Cross-talk between NKT and γδT cells has been reported (Paget et al., 2012). For example, γδT cells were more abundant in the livers of Jα18 KO mice and were also the primary producers of IL-17 in a mouse model of autoimmune hepatitis. Moreover, administration of anti-γδT cell receptor antibody to KO mice abolished disease, including IL-17 production (Nishio et al., 2015). As a conclusion, authors suggest a defending role for iNKT cells, a pathologic role for γδT cells, and a link between these cells in the presence of illness. Results from other investigators indicated that liver inflammation mediated by TRL3 agonist can be resolute by activation of γδT by NKT cells (Gardner et al., 2010). Conversely, in a model of hepatitis Tγδ17 have been reported as negative regulators of NKT cell activation (Zhao et al., 2011). Finally, airway hyperresponsiveness can be heightened through the concomitant action of NKT and Vg1^+^γδT cells (Jin et al., 2007). Thus, γδT cells are capable of positively or negatively adjust NKT cell response in relation to the tissue analyzed and the subset of γδT cells triggered. Here we show that the activation of iNKT cells with α-GalCer abrogated IL-17 production and down regulated Tγδ17 in MLN, thus preventing gut inflammation and ReA induced by *Salmonella* enterocolitis.

In summary, we utilized an animal model to investigate the participation of iNKT in the pathogenesis of ReA triggered by *Salmonella* enterocolitis. We found that the activation of iNKT by α-GalCer protects the host from the inflammatory response induced during *Salmonella* enterocolitis. α-GalCer treatment inhibited the production of IL-17, averted intestinal inflammation and prevented ReA. Abrogation of IL-17 production was related to the down regulation of IL-17 producing γδT cells –rather Th17 cells.

## Author contributions

MN and SS: equally contributed to this manuscript, conceived carried out experiments, and wrote it; AM: carried out and analyzed data; MG and MA: analyzed and interpreted the data; GB: provided assistance in performing flow cytometry assays; MC: was responsible for the overall study design and took responsibility for writing the manuscript.

### Conflict of interest statement

The authors declare that the research was conducted in the absence of any commercial or financial relationships that could be construed as a potential conflict of interest. The reviewer NC and handling Editor declared their shared affiliation.

## Abbreviations

ReA: reactive arthritis
EC: enterocolitis
Inkt: Invariant natural killer T
TCR: T cell receptor
Jα18^−/−^ KO: Jα18-knocked out
qPCR: quantitative Polymerase Chain Reaction
γδT: gamma delta T cells
IL17-γδTcells: IL-17 producers gamma delta T cells
α-GalCer: α-*galactosylceramide*
MLN: mesenteric lymph node.
