# Supplementary material for: Activation of iNKT Cells Prevents Salmonella-Enterocolitis and Salmonella-Induced Reactive Arthritis by Downregulating IL-17-Producing γδT Cells
Source: Front Cell Infect Microbiol. 2017 Sep 8;7:398. doi: 10.3389/fcimb.2017.00398 (PMC5596086; doi:10.3389/fcimb.2017.00398)
Supplement: Supplementary file 1 [file Table1.DOC]

Supplementary Table 1. Primers used in this study.

| **Target** | **Sequence** (5’→3’) ***^a^*** | **Accession number** | **Ref.** |
| --- | --- | --- | --- |
| IL-17A | GCTCCAGAAGGCCCTCAGA (F) | U43088 | Godinez et al., 2009 |
|  | AGCTTTCCCTCCGCATTGA (R) |  |  |
| Vα14 Jα281 TCR | CTAAGCACAGCACGCTGCACA (F) | AY158219 | Kim et al., 2005 |
|  | AGGTATGACAATCAGCTGAGTCCC (R) |  |  |
| IFN-γ | TGGCTGTTTCTGGCTGTTACT (F) | NM_008337.4 | This work |
|  | GGTTGTTGACCTCAAACTTGG (R) |  |  |
| 18S rRNA | AACACGGGAAACCTCACCC (F) | GU372691 | Nur et al., 2013 |
|  | CCACCAACTAAGAACGGCCA (R) |  |  |

Primers were purchased from Invitrogen Inc. and were designed according to the DNA sequence information available for *Mus musculus* (*M. musculus* blast server BLAST Server Database at [www.sanger.ac.uk](http://www.sanger.ac.uk/)). *^a^* F, forward primer; R, reverse primer.
